# Association of Social Determinants of Health With Hospital Readmission and Mortality: A Prospective Cohort Study

**DOI:** 10.3928/24748307-20240702-01

**Published:** 2024-10

**Authors:** Amanda S. Mixon, Kathryn Goggins, Samuel Nwosu, Yaping Shi, Jonathan S. Schildcrout, Kenneth A. Wallston, Gabriela Leon-Perez, Frank E. Harrell, Susan P. Bell, Lindsay S. Mayberry, Eduard E. Vasilevskis, John F. Schnelle, Russell L. Rothman, Sunil Kripalani, for the Vanderbilt Inpatient Cohort Study

## Abstract

**Background:**

The relative contributions of common patient-reported social determinants of health on 30- and 90-day post-discharge outcomes among patients with acute coronary syndromes (ACS) is unclear.

**Objective:**

The aim of this article is to examine the independent associations of social determinants with readmission or death, accounting for medical history.

**Methods:**

Participants included adults who were hospitalized with ACS at an academic medical center. Domains measured were social support, health literacy/numeracy, and socioeconomic status (SES) (including education and difficulty paying bills). We employed multivariable Cox proportional hazard models to study associations with time to all-cause readmission or death, up to 30 or 90 days after discharge, and adjusted for demographics and medical history (prior admissions and Elixhauser comorbidity index).

**Key Results:**

Among 1,168 patients with ACS and no history of heart failure, more prior admissions, and higher comorbidity index (the medical history domain) were associated with higher rates of 30- and 90-day readmission or death (domain *p* values <.01 and <.0001, respectively). The social support domain was not associated with outcomes. Higher health literacy and numeracy were associated with lower rates of 30- and 90-day readmission or death (domain *p* values .016 and .002, respectively). Higher education and less difficulty paying bills (the SES domain) was marginally associated with lower rates of 90-day readmission or death (domain, *p* = .052).

**Conclusions:**

In addition to medical history, the domain of health literacy and numeracy was independently associated with readmission or death of patients with ACS during the 90 days after hospital discharge. [***HLRP: Health Literacy Research and Practice*. 2024;8(4):e212–e223.**]

## Abstract (plain language summary)

**Plain Language Summary: **In this study of patients admitted to the hospital with heart conditions, we asked them about their social support, how they understood health information (health literacy), economic status, education, and difficulty paying bills. We found that patients with higher health literacy had less returns to the hospital or died less frequently within 30 and 90 days of their initial stay.

The postdischarge period after hospitalization is a vulnerable time for patients, when increased self-management requirements are common (Guo & Harris, 2016; Wiggins et al., 2013). The self-management tasks may be challenging for patients discharged with high-risk diagnoses such as acute coronary syndromes (ACS). Additionally, medication regimens may change significantly during hospitalization (Harris et al., 2013; Medication safety in transitions of care., 2019). Prior to discharge, patients often receive discharge education in a hurried manner, seldom tailored to their level of health literacy (Blake et al., 2010; Coleman, 2003; Coleman et al., 2013; Daliri et al., 2019; Eibergen et al., 2018). Moreover, once patients leave the hospital, their ability to self-manage may also be affected by the amount of social support they receive or resources available based on their socioeconomic status (SES) (Hardman, 2020; King et al., 2023; Salyer et al., 2012).

A wide array of social determinants of health can impact health outcomes, yet these factors are often not assessed or attended to during hospitalization (Adler et al., 2016; Horwitz et al., 2020). Low health literacy, numeracy, and social support have been associated with poor outcomes in diseases requiring self-management such as coronary artery disease (Aburadwan & Hayajneh, 2024; Beauchamp et al., 2022; Berkman et al., 2011). However, in hospital-based studies, the relationship between health literacy and readmissions has been inconsistent (Ghisi et al., 2018; Kanejima et al., 2022; McNaughton, Cawthon, et al., 2015; Sterling et al., 2018). SES, including income, employment, and education, can affect an individual's adherence to the recommended medical regimen and has been linked with readmissions and mortality (Chiu et al., 2022; Khera et al., 2017). Despite recognition of the importance of these individual risk factors, they frequently co-exist and have not been examined together to determine their relative and independent contributions to postdischarge outcomes for patients with cardiovascular disease.

Our objective was to examine the independent associations of health literacy, numeracy, social support, and SES with poor outcomes post-discharge, specifically unplanned readmission, or death, among patients hospitalized for ACS. We hypothesized that patients who had lower health literacy, numeracy, social support, or SES would have higher rates of readmission or death during the first 30- or 90-days postdischarge, after adjustment for demographic characteristics, medical comorbidities, and prior utilization.

## Methods

### Study Setting and Design

The Vanderbilt Inpatient Cohort Study (VICS) was a prospective longitudinal study of the impact of patient, social, and medical factors on post-discharge health outcomes such as quality of life, unplanned hospital utilization, and mortality in adults with ACS or acute decompensated heart failure (ADHF). The rationale and design of VICS are detailed elsewhere (Meyers et al., 2014). Briefly, the framework that guided this study posits that demographic factors such as age, race, ethnicity, and socioeconomic status may influence health status, social support, and health literacy. As a result, those three factors may affect how patients interact with health systems, health care providers, and their disease self-management. Taken together, all factors may impact health outcomes such as functional status, health-related quality of life, unplanned health care utilization, and mortality. The study was approved by the Vanderbilt University Medical Center Institutional Review Board.

### Participants

Research staff screened patients admitted to Vanderbilt University Hospital who presented with symptoms consistent with ADHF or an intermediate or high likelihood of ACS. A study hospitalist or cardiologist confirmed the diagnosis by reviewing the electronic health record (EHR). Exclusion criteria included: age younger than 18 years, non-English speaker, hearing or vision impairment, unstable psychiatric illness, delirium, low likelihood of follow-up (e.g., no reliable telephone number), on hospice, too ill to complete an interview, or prior enrollment in the study. Once patients with cardiovascular disease agreed to participate, written informed consent was obtained during hospitalization. Enrollment spanned from October 2011 to December 2015. Herein, we report on results for participants with ACS. An analysis of participants with ADHF has been reported previously (Sterling et al., 2018).

During participants' hospitalization, research assistants administered a 45-minute baseline interview at the bedside. Baseline measures included demographic characteristics such as age, gender, and self-reported race.

### Measures of Medical History

Participants reported their number of hospitalizations in the prior year. Additionally, a comorbidity index, based on 30 conditions identified by Elixhauser, was calculated from billing codes from the index hospitalization and prior utilization (van Walraven et al., 2009). This single numerical index calculated from the 30 Elixhauser comorbidities had equivalent discrimination when compared to the inclusion of the 30 variables individually (c-statistic 0.763 vs 0.760, respectively), while conserving degrees of freedom.

### Measures of Social Support and Marital and Living Status

We determined social support of family and friends prior to hospitalization using measures that characterize instrumental as well as emotional support. Instrumental support is the tangible support received from other people, their informal support network, and unmet personal needs (Schultz et al., 2022). For instrumental support, we drew questions from: (1) the Health and Retirement Study, which quantified the number of friends and family members with whom they had close relationships (Juster & Suzman, 1995); and (2) the Midlife Development in the United States (MIDUS), which quantified the number and frequency of contacts, level of support from friends, families, and neighbors (Rossi, 2004). To assess emotional support (e.g. someone to listen to them, give advice, show love and affection), we drew questions from the ENRICHD Social Support Inventory (ESSI), which has a Cronbach α of 0.86 and Pearson correlation coefficient (ρ) of 0.62 with the Perceived Social Support Scale (Berkman et al., 2003; Blumenthal et al., 1987; The ENRICHD Investigators, 2000).

Additionally, we asked participants their marital status and whether they lived alone. From their responses we created home status, a two-level categorical variable: married or living with someone versus not married and living alone.

### Measures of Health Literacy and Numeracy

Health literacy is “the degree to which individuals have the capacity to obtain, process, and understand basic health information and services needed to make appropriate health decisions” (National Heart, Lung, and Blood Institute, 2000). During the baseline interview, we assessed subjective health literacy using the 3-item Brief Health Literacy Screen (BHLS), scored on a 5-point Likert scale (Chew et al., 2004). We report the sum on a scale ranging from 3 to 15 points, with higher scores indicating higher health literacy. In the hospital setting, the Cronbach α is 0.79, and the Pearson correlation coefficient (ρ) was 0.48 when compared to s-TOFHLA (Wallston et al., 2014).

Numeracy is “the ability to use and understand numbers in daily life” (Golbeck et al., 2005). We measured numeracy using the 3-item Subjective Numeracy Scale (SNS-3). This self-reported measure captures participants' quantitative abilities with numerical data and preferences for numerical information. The SNS-3 is reported as a mean on a scale of 1 to 6. The SNS-3's Cronbach's α for internal reliability ranges from 0.67 to 0.86 for 7 study samples, and it correlates very highly with the original longer measure, the SNS-8 (range of *ρ* = 0.89 − 0.95) (Fagerlin et al., 2007; McNaughton, Cavanaugh, et al., 2015).

### Measures of SES

Participants reported their highest level of education achieved and employment status. We assessed financial strain with a question: “How difficult is it for you (and your family) to pay your monthly bills?” Responses were reverse coded and ranged from 1, *very difficult*, to 4, *not at all difficult* (Osborn et al., 2017).

### Outcome Measures

The primary outcomes were a composite endpoint of all-cause readmission or death, assessed as time to event during the first 30 or 90 days after discharge. Readmission included hospitalization in any acute care hospital. Outcomes were compiled from the Vanderbilt EHR, participant report during follow-up phone calls, and a complete review of outside hospital records. Participants who died during the index hospitalization (*n* = 23) were excluded from these analyses because they did not enter the follow-up period.

### Analysis

We describe the VICS participants with ACS using proportions for categorical variables and percentiles (i.e., 25th, 50th, 75th) for quantitative variables. To test for unadjusted, covariate associations with readmission/death at 30 and 90 days, we used Pearson Chi-Square test and Wilcoxon Rank-Sum test for the categorical and quantitative variables, respectively.

For primary analyses, we conducted multivariable Cox proportional hazards regression analyses to investigate associations between participant characteristics and the two dependent variables: time to readmission or death up to 30 days and time to readmission or death up to 90 days after discharge. We report hazard ratios, confidence intervals, and *p* values to describe adjusted associations with outcomes. Specifically, we report hazard ratios and confidence intervals associated with interquartile range changes in independent, continuous variables.

Guided by the study's conceptual framework, and in addition to examining individual independent variable associations with outcomes, we report outcome associations with five independent variable domains--demographics, medical history, social support, health literacy/numeracy, and SES. We conducted domain-outcome association tests using likelihood ratio tests (LRT) with degrees of freedom equal to the number of parameters estimated for the domain. For these domain-specific associations, the LRT compares a model that excludes all variables in the domain to a model that includes them all, in both cases adjusting for all other independent variables.

To further characterize the overall associations of the medical history, social support, health literacy/numeracy, and SES domains with time to readmission or death at 30 and 90 days, we examined association with simultaneous independent variable changes within each domain. Specifically, we examined time to readmission or death associations as follows: (1) for the medical history domain, a simultaneous 1 additional prior admission within 12 months and a 4.5 point increase in Elixhauser score; (2) for the social support domain, a 3 point increase in ESSI and living with someone versus living alone; (3) for the health literacy/numeracy domain, a 2.5 point increase in BHLS and 1 point increase in subjective numeracy; (4) for the SES domain, a 2 year increase in education and a 1 category increase in paying bills score (i.e. when reverse coded it is an increased ease of paying bills). The simultaneous changes in the quantitative variables correspond to approximately one-half interquartile range changes.

To avoid case-wise deletion of records with missing covariates we conducted multiple imputation with five imputation samples using a predictive mean matching algorithm (Harrell, 2016; Rubin & Schenker, 1991). Missingness rates were low. We conducted all analyses in R version 4.1.3 (R Core Team, 2022) with packages rms (Harrell, 2021) and survival (Therneau, 2021), and used 0.05-level significance tests.

## Results

**Figure 1.**
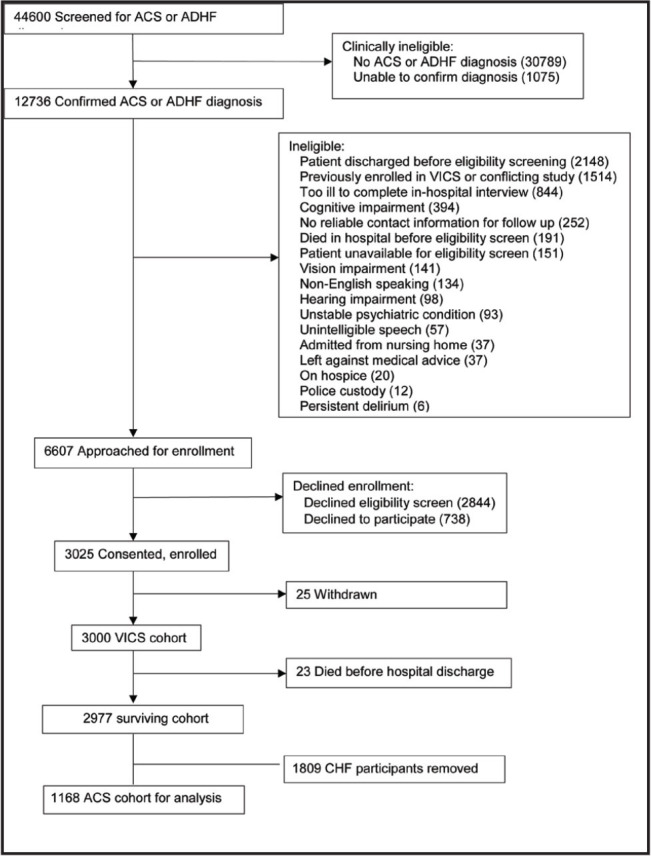
Study flow diagram. The authors recruited, consented, and enrolled 3,000 patients with ACS or ADHF to the VICS cohort, of which 1,168 with ACS were analyzed. ACS = acute coronary syndrome, ADHF = acute decompensated heart failure, VICS = Vanderbilt Inpatient Cohort Study.

**Figure 1** displays the study flow diagram. Of 44,600 patient charts screened, 12,736 (28.6%) had confirmed diagnoses of ACS and/or ADHF. Of 12,736 patients, nearly 30% (3,763) met all eligibility criteria, 80% of whom enrolled. Of the 3,000 participants in the cohort, 2,977 participants were discharged alive. For these analyses, 1168 (39%) had ACS with no prior diagnosis of congestive heart failure. **Tables 1** and **2** describe the ACS sample stratified by 30-day and 90-day death/readmission status, respectively. Overall, participants had a median age of 60 years and a median of 14 years of education (**Table 1**). There were 113 (9.7%) participants who were Black or African American, Asian, Native Hawaiian or other Pacific Islander, American Indian or Alaska Native, other underrepresented races, and unknown race, and 445 (38%) were female. Approximately 10% of participants (*n* = 118) were readmitted or died within 30 days after discharge; 17% (*n* = 206) were readmitted or died within 90 days (**Table 2**). In unadjusted tests, we observed statistically significant differences in the distributions of income, employment status, number of admissions in the past 12 months, health literacy, numeracy and difficulty paying bills, between participants who were readmitted or died at 30 and 90 days. Additionally, at 90 days there were significant differences in education, Elixhauser score, and social support.

**Table 1:**
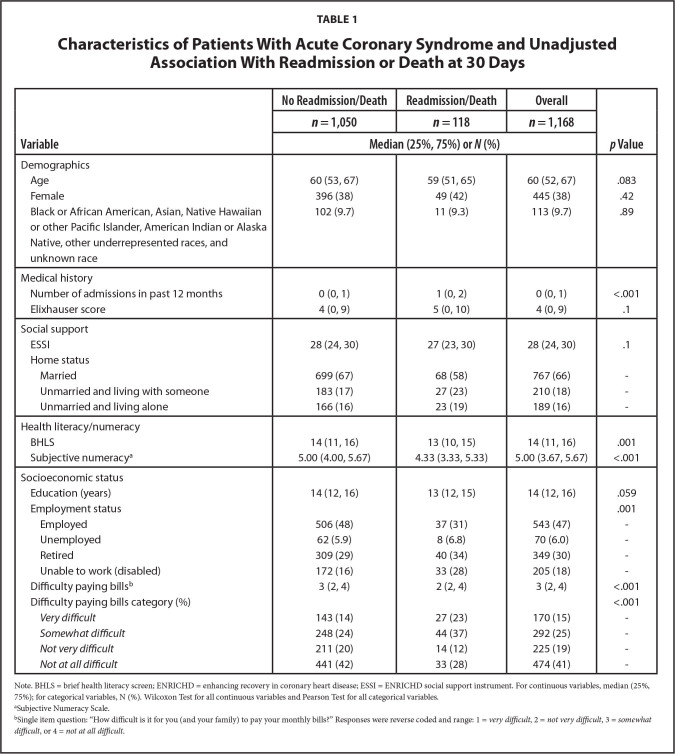
Characteristics of Patients With Acute Coronary Syndrome and Unadjusted Association With Readmission or Death at 30 Days

**Variable**	**No Readmission/Death**	**Readmission/Death**	**Overall**	***p* Value**

	***n*= 1,050**	***n*= 118**	***n*= 1,168**	

	**Median (25%, 75%) or *N*(%)**			

Demographics				
Age	60 (53, 67)	59 (51, 65)	60 (52, 67)	.083
Female	396 (38)	49 (42)	445 (38)	.42
Black or African American, Asian, Native Hawaiian or other Pacific Islander, American Indian or Alaska Native, other underrepresented races, and unknown race	102 (9.7)	11 (9.3)	113 (9.7)	.89

Medical history				
Number of admissions in past 12 months	0 (0, 1)	1 (0, 2)	0 (0, 1)	<.001
Elixhauser score	4 (0, 9)	5 (0, 10)	4 (0, 9)	.1

Social support				
ESSI	28 (24, 30)	27 (23, 30)	28 (24, 30)	.1
Home status				
Married	699 (67)	68 (58)	767 (66)	-
Unmarried and living with someone	183 (17)	27 (23)	210 (18)	-
Unmarried and living alone	166 (16)	23 (19)	189 (16)	-

Health literacy/numeracy				
BHLS	14 (11, 16)	13 (10, 15)	14 (11, 16)	.001
Subjective numeracy^a^	5.00 (4.00, 5.67)	4.33 (3.33, 5.33)	5.00 (3.67, 5.67)	<.001

Socioeconomic status				
Education (years)	14 (12, 16)	13 (12, 15)	14 (12, 16)	.059
Employment status				.001
Employed	506 (48)	37 (31)	543 (47)	-
Unemployed	62 (5.9)	8 (6.8)	70 (6.0)	-
Retired	309 (29)	40 (34)	349 (30)	-
Unable to work (disabled)	172 (16)	33 (28)	205 (18)	-
Difficulty paying bills^b^	3 (2, 4)	2 (2, 4)	3 (2, 4)	<.001
Difficulty paying bills category (%)				<.001
* Very difficult*	143 (14)	27 (23)	170 (15)	-
*Somewhat difficult*	248 (24)	44 (37)	292 (25)	-
*Not very difficult*	211 (20)	14 (12)	225 (19)	-
*Not at all difficult*	441 (42)	33 (28)	474 (41)	-

Note. BHLS = brief health literacy screen; ENRICHD = enhancing recovery in coronary heart disease; ESSI = ENRICHD social support instrument. For continuous variables, median (25%, 75%); for categorical variables, N (%). Wilcoxon Test for all continuous variables and Pearson Test for all categorical variables.

a Subjective Numeracy Scale.

b Single item question: “How difficult is it for you (and your family) to pay your monthly bills?” Responses were reverse coded and range: 1 = *very difficult*, 2 = *not very difficult*, 3 = *somewhat difficult*, or 4 = *not at all difficult*.

**Table 2:**
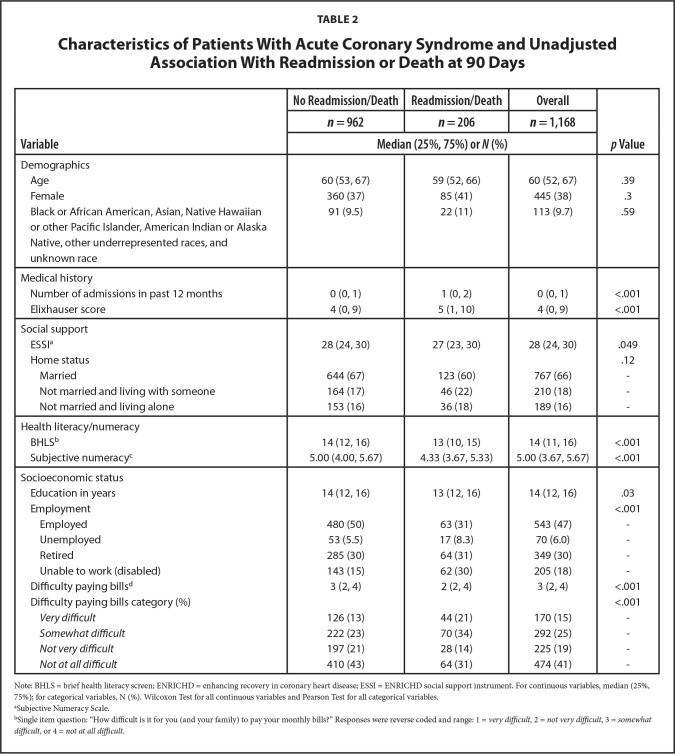
Characteristics of Patients With Acute Coronary Syndrome and Unadjusted Association With Readmission or Death at 90 Days

**Variable**	**No Readmission/Death**	**Readmission/Death**	**Overall**	***p* Value**

	***n* = 962**	***n* = 206**	***n* = 1,168**	

	**Median (25%, 75%) or *N* (%)**			

Demographics				
Age	60 (53, 67)	59 (52, 66)	60 (52, 67)	.39
Female	360 (37)	85 (41)	445 (38)	.3
Black or African American, Asian, Native Hawaiian	91 (9.5)	22 (11)	113 (9.7)	.59
or other Pacific Islander, American Indian or Alaska Native, other underrepresented races, and unknown race				

Medical history				
Number of admissions in past 12 months	0 (0, 1)	1 (0, 2)	0 (0, 1)	<.001
Elixhauser score	4 (0, 9)	5 (1, 10)	4 (0, 9)	<.001

Social support				
ESSI^a^	28 (24, 30)	27 (23, 30)	28 (24, 30)	.049
Home status				.12
Married	644 (67)	123 (60)	767 (66)	-
Not married and living with someone	164 (17)	46 (22)	210 (18)	-
Not married and living alone	153 (16)	36 (18)	189 (16)	-

Health literacy/numeracy				
BHLS^b^	14 (12, 16)	13 (10, 15)	14 (11, 16)	<.001
Subjective numeracy^c^	5.00 (4.00, 5.67)	4.33 (3.67, 5.33)	5.00 (3.67, 5.67)	<.001

Socioeconomic status				
Education in years	14 (12, 16)	13 (12, 16)	14 (12, 16)	.03
Employment				<.001
Employed	480 (50)	63 (31)	543 (47)	-
Unemployed	53 (5.5)	17 (8.3)	70 (6.0)	-
Retired	285 (30)	64 (31)	349 (30)	-
Unable to work (disabled)	143 (15)	62 (30)	205 (18)	-
Difficulty paying bills^d^	3 (2, 4)	2 (2, 4)	3 (2, 4)	<.001
Difficulty paying bills category (%)				<.001
*Very difficult*	126 (13)	44 (21)	170 (15)	-
*Somewhat difficult*	222 (23)	70 (34)	292 (25)	-
*Not very difficult*	197 (21)	28 (14)	225 (19)	-
*Not at all difficult*	410 (43)	64 (31)	474 (41)	-

Note: BHLS = brief health literacy screen; ENRICHD = enhancing recovery in coronary heart disease; ESSI = ENRICHD social support instrument. For continuous variables, median (25%, 75%); for categorical variables, N (%). Wilcoxon Test for all continuous variables and Pearson Test for all categorical variables.

a Subjective Numeracy Scale.

b Single item question: “How difficult is it for you (and your family) to pay your monthly bills?” Responses were reverse coded and range: 1 = *very difficult*, 2 = *not very difficult*, 3 = *somewhat difficult*, or 4 = *not at all difficult*.

**Figure 2.**
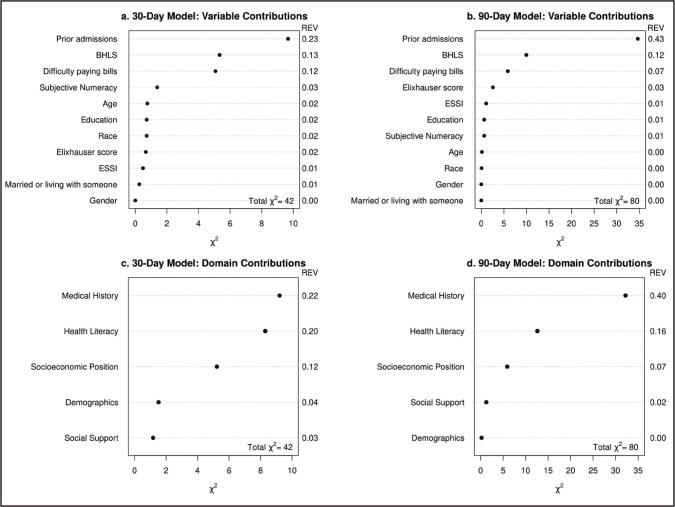
Individual variable and domain contributions to the explained variation in the time to readmission or death models. We report the Chi-square statistics and the relative explained variation (REV). REV was calculated by the Chi-square statistic contributed by each variable and each domain divided by the total Chi-square for the whole model.

**Table 3** shows results from the multivariable Cox models for the 30- and 90-day outcomes. We display estimated hazard ratios, 95% confidence intervals and *p* values for individual variable associations with instantaneous rates of readmission or death within 30 and 90 days of discharge. We also report likelihood ratio test *p* values corresponding to outcome associations with key domains (i.e., groups of variables) and hazard ratio and 95% confidence interval estimates to characterize outcome associations with simultaneous changes in multiple variables within the social support, health literacy, and SES domains. For the 30-day analysis, prior admissions were positively associated with readmission or death (*HR* = 1.32, 95% confidence interval [CI]: 1.11 − 1.58 per 2 prior admissions within the last year). The health literacy/numeracy domain was significantly associated with readmission or death (domain *p* = 0.016). A simultaneous increase in BHLS (2.5 points) and subjective numeracy (1 point) was associated with a hazard ratio of 0.76 (95% CI: 0.63 – 0.91). We did not observe an association between the social support or SES domains and readmission or death at 30 days after discharge. **Figure 2** shows individual variable and domain associated Chi-Square statistics as well as a measure of relative explained variability. We observed that the health literacy/numeracy domain explains far more variability than either of the socioeconomic position and social support domains (**Figures 2A and 2C**), although medical history explained the most variability in time to readmission/death by 30 days post discharge.

**Table 3:**
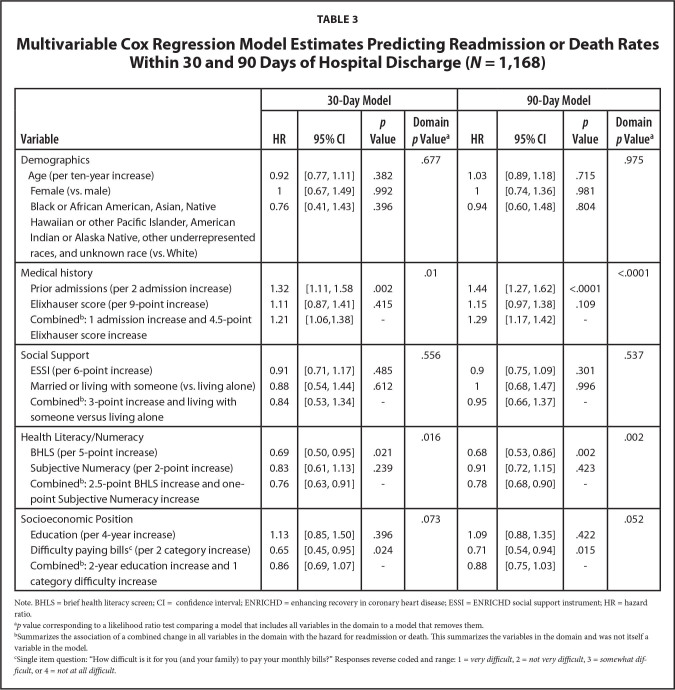
Multivariable Cox Regression Model Estimates Predicting Readmission or Death Rates Within 30 and 90 Days of Hospital Discharge (*N* = 1,168)

**Variable**	**30-Day Model**				**90-Day Model**			

	**HR**	**95% CI**	***p* Value**	**Domain *p* Value^a^**	**HR**	**95% CI**	***p* Value**	**Domain *p* Value^a^**

Demographics				.677				.975
Age (per ten-year increase)	0.92	[0.77, 1.11]	.382		1.03	[0.89, 1.18]	.715	
Female (vs. male)	1	[0.67, 1.49]	.992		1	[0.74, 1.36]	.981	
Black or African American, Asian, Native Hawaiian or other Pacific Islander, American Indian or Alaska Native, other underrepresented races, and unknown race (vs. White)	0.76	[0.41, 1.43]	.396		0.94	[0.60, 1.48]	.804	

Medical history				.01				<.0001
Prior admissions (per 2 admission increase)	1.32	[1.11, 1.58	.002		1.44	[1.27, 1.62]	<.0001	
Elixhauser score (per 9-point increase)	1.11	[0.87, 1.41]	.415		1.15	[0.97, 1.38]	.109	
Combined^b^: 1 admission increase and 4.5-point	1.21	[1.06,1.38]	-		1.29	[1.17, 1.42]	-	
Elixhauser score increase								

Social Support				.556				.537
ESSI (per 6-point increase)	0.91	[0.71, 1.17]	.485		0.9	[0.75, 1.09]	.301	
Married or living with someone (vs. living alone)	0.88	[0.54, 1.44]	.612		1	[0.68, 1.47]	.996	
Combined^b^: 3-point increase and living with someone versus living alone	0.84	[0.53, 1.34]	-		0.95	[0.66, 1.37]	-	

Health Literacy/Numeracy				.016				.002
BHLS (per 5-point increase)	0.69	[0.50, 0.95]	.021		0.68	[0.53, 0.86]	.002	
Subjective Numeracy (per 2-point increase)	0.83	[0.61, 1.13]	.239		0.91	[0.72, 1.15]	.423	
Combined^b^: 2.5-point BHLS increase and one-point Subjective Numeracy increase	0.76	[0.63, 0.91]	-		0.78	[0.68, 0.90]	-	

Socioeconomic Position				.073				.052
Education (per 4-year increase)	1.13	[0.85, 1.50]	.396		1.09	[0.88, 1.35]	.422	
Difficulty paying bills^c^ (per 2 category increase)	0.65	[0.45, 0.95]	.024		0.71	[0.54, 0.94]	.015	
Combined^b^: 2-year education increase and 1 category difficulty increase	0.86	[0.69, 1.07]	-		0.88	[0.75, 1.03]	-	

Note. BHLS = brief health literacy screen; CI = confidence interval; ENRICHD = enhancing recovery in coronary heart disease; ESSI = ENRICHD social support instrument; HR = hazard ratio.

a *p* value corresponding to a likelihood ratio test comparing a model that includes all variables in the domain to a model that removes them.

b Summarizes the association of a combined change in all variables in the domain with the hazard for readmission or death. This summarizes the variables in the domain and was not itself a variable in the model.

c Single item question: “How difficult is it for you (and your family) to pay your monthly bills?” Responses reverse coded and range: 1 = *very difficult*, 2 = *not very difficult*, 3 = *somewhat difficult*, or 4 = *not at all difficult*.

The 90-day Cox proportional hazard model was similar to the 30-day model; however, due to the larger number of events by 90 days, confidence interval widths are smaller. Prior admissions were significantly associated with readmission or death (*HR* = 1.44, 95% CI: 1.27 − 1.62 per 2 prior admissions), and higher BHLS scores were associated with lower rates of readmission or death (*HR* = 0.68, 95% CI: 0.53 − 0.86 per 5-point change). Overall, the medical history and health literacy/numeracy domains were associated with 90-day readmission or death (domain, *p* < .0001 and *p* < .002, respectively), though the SES domain was marginally associated (domain, *p* = .052). A simultaneous increase in BHLS (2.5 points) and subjective numeracy (1 point) scores was associated with a hazard ratio of 0.78 (95% CI: 0.68 – 0.90), and a simultaneous increase in education (2 years) and difficulty paying bills (1 category increase) was associated with a hazard ratio of 0.88 (95% CI: 0.75 − 1.03), which was marginally significant. We observed in **Figure 2** that medical history explained the most variability in time to readmission/death (**Figures 2B and 2D**), but that the health literacy/numeracy domain explained far more variability than socioeconomic position and social support domains.

## Discussion

In this large cohort study of participants with ACS, we found complex associations between social determinants of health and post-discharge outcomes. After adjustment for prior health care utilization and medical comorbidities, the domain of health literacy/numeracy was independently associated with hospital readmission or death through 90 days post-discharge. SES was associated with outcomes in unadjusted analyses but was not statistically significant (*p* = 0.052 and *p* = .072) in adjusted analyses. Surprisingly, social support, measured in a variety of ways, was unrelated to readmission or death when adjusting for other factors. This research demonstrates that, when these common social determinants of health are considered together, health literacy/numeracy has the most dominant and consistent effect on the post-discharge outcomes examined.

Our findings have implications for the ongoing national dialogue on how social determinants of health affect hospital readmission rates and which factors may be most important to measure and address in hospitalized patients. Models for readmission vary in their predictive ability, depending on which types of variables are included. In our prior systematic review, we reported the majority of models used comorbidities and prior utilization to predict readmission with modest discrimination (c-statistics 0.6–0.77) (Kansagara et al., 2011). Interestingly, models that utilized administrative data as well as social determinants of health have demonstrated better predictive abilities. Yet, thus far there has been no agreement on what the most predictive social determinants of health are (Joynt et al., 2017). No published models have included health literacy, which our findings suggest is an important omission.

We found an independent association between health literacy/numeracy and outcomes among participants with ACS. Previously using data on participants from the same cohort who had acute decompensated heart failure (ADHF), we had also found health literacy was associated with greater medical complexity, including being admitted for ADHF for the index hospitalization and more hospitalizations in the prior year (Mayberry et al., 2018). Our prior finding supports the notion that health literacy has already exerted an effect on medical complexity which leads to readmissions and/or death. Moreover, the health literacy/numeracy domain is thought to affect post-discharge outcomes through effects on patients' self-management of their medical conditions. For example, medication use or ability to detect and respond to warning signs of clinical decompensation. The current results are consistent with other investigations which have demonstrated associations between health literacy and readmission rates for ACS (Kanejima et al., 2022; Mitchell et al., 2012). Further investigations into the mechanism by which health literacy/numeracy impacts self-management activities and risk for readmission are warranted for patients with cardiovascular disease. A few potential mediators particularly warranting study, which are known to be more common among patients with low health literacy/numeracy, are medication errors, medication nonadherence (particularly unintentional nonadherence), and missed follow-up appointments (Knolhoff et al., 2016; Lindquist et al., 2012; Mixon et al., 2014).

Our hypothesis that a=dverse post-discharge outcomes would be related to poorer social support was noted in unadjusted results at 90 days, but its effect was attenuated by other factors in the adjusted models, which was surprising. Being a widow(er) or nonmarried have also been associated previously with increased risk of mortality in ACS (Hadi Khafaji et al., 2012; Marcus et al., 2019). Perhaps the measures we utilized did not adequately capture the challenges patients with ACS face with regard to social support.

We examined the overall direct effects of the specified social determinants of health on readmissions and mortality. From the same cohort, we found that health literacy indirectly affects 1-year mortality via worse health behaviors, lower perceived health competence, and more medical complexity, including comorbidities and being admitted for ADHF, but not via social support (Mayberry et al., 2018). In the present analysis, we did not examine moderating variables, such as severity of illness; therefore, it may be premature to conclude that social support is not predictive of post-discharge outcomes. Among patients with severe illness, prognosis may be determined primarily by physiologic factors, whereas in patients with less severe illness, health literacy and numeracy may play a greater role in prognosis through their effect on self-management. At present, we maintain that assessing social determinants of health for patients is critical to providing appropriate and equitable care (Brandt et al., 2023), and they may manifest in other ways not measured here, ranging from patient satisfaction to medication safety. For example, health literacy sensitive materials should be used for patient discharge education as a commonsense approach (Glick et al., 2019; Wiggins et al., 2013). Additionally, social services and care transitions teams should determine hospitalized patients' social support to maximize success after discharge.

## Study Strengths and Limitations

Our study's strengths include a large sample size including 38% women, minimal missing or incomplete data, multiple measures of health literacy and social support, and outcome assessment including data from any reported hospital. In fact, for the overall study 29% of first readmissions captured were to another facility. In contrast, we must consider the potential limitations. The participants came from one referral hospital, though they originated from more than 20 states. We excluded 2% of eligible patients from enrollment who lacked a stable phone number for follow up. These individuals might have experienced greater impact of social determinants of health on outcomes, so excluding them may bias our results toward the null. We focused on one health condition, ACS, so it is unclear how generalizable our results are to a wider hospitalized population. We had time horizons of 30- and 90-days follow-up for our readmissions and mortality data; however, these time points are relevant to current policies regarding readmission penalties and episodes of care. As with many psychosocial factors, mediating factors may influence the effect of social support and health literacy on outcomes (e.g. adherence, self-management), which we did not examine. We did not include environmental or neighborhood SES factors. Finally, the study relied on data from participant self-report, which is subject to reporting bias, although we used well-validated instruments, and patient-level social data are superior to community-level data (Kostelanetz et al., 2021).

## Conclusion

In this cohort of participants hospitalized with ACS, we determined that health literacy/numeracy was significantly associated with readmission or mortality in the 30-to-90-day postdischarge period, when adjusting for other factors. Other social determinants of health were not consistently related to outcomes. Health literacy/numeracy warrant greater attention amidst growing national efforts to screen social determinants of health in hospital settings.
